# P06 The burden of surgical site infections in vascular surgery: epidemiology, microbiology and 30 day mortality in North Wales, United Kingdom

**DOI:** 10.1093/jacamr/dlaf230.013

**Published:** 2025-12-04

**Authors:** Armia Sargious, Faisal M Shaikh, Muhammad Mohsin, Buddug Ecklay, Abigail Williams, Sudhara Budagoda, Mohan Devbhandari, Laszlo Papp

**Affiliations:** Vascular Surgery Department, North Wales, Betsi Cadwaladr University Health Board, UK; Vascular Surgery Department, North Wales, Betsi Cadwaladr University Health Board, UK; Vascular Surgery Department, North Wales, Betsi Cadwaladr University Health Board, UK; Vascular Surgery Department, North Wales, Betsi Cadwaladr University Health Board, UK; Vascular Surgery Department, North Wales, Betsi Cadwaladr University Health Board, UK; Vascular Surgery Department, North Wales, Betsi Cadwaladr University Health Board, UK; Vascular Surgery Department, North Wales, Betsi Cadwaladr University Health Board, UK

## Abstract

**Background:**

Surgical site infections (SSIs) represent a major cause of morbidity in vascular surgery, with incidence rates demonstrating significant variation based on procedure type and anatomical location. While clean vascular procedures carry a 2%–4% SSI risk, this increases substantially for cases involving groin or lower limb incisions. The clinical consequences are severe, including prolonged hospitalization, higher mortality, significant healthcare expenditures and increased re-intervention requirements.

**Objectives:**

In this study we examine the incidence, microbiological characteristics and clinical outcomes of SSIs at a vascular tertiary referral centre, addressing a notable gap in contemporary United Kingdom (UK) vascular literature. Our findings aim to inform improved prevention protocols and management strategies for these potentially devastating complications.

**Methods:**

We conducted a retrospective analysis of all patients admitted in vascular surgery, from 1 June 2020 to 31 July 2021 who developed SSIs post-admission at our institute in North Wales (UK). Our centre is the vascular hub serving all the North Wales hospitals. Data were collected on demographics, procedure type, infection site, microbial isolates, length of stay (LOS), mortality and re-intervention rates.

**Results:**

During the study period 943 patients were admitted in our department. 56 patients (5.9%) developed SSIs, with 86% occurring following emergency procedures. The groin (41%) and major amputation stumps (29%) were the most common infection sites. High-risk patients were elderly males undergoing emergency revascularization. Gram-negative organisms (coliforms, *Pseudomonas aeruginosa*) were the most predominant causes, followed by Enterococcus spp. and *Staphylococcus aureus*. Polymicrobial infections (35.7%) were associated with early onset (mean 5 days) and prolonged LOS (mean 44 days). Enterococcus infections had the latest onset (mean 16 days) and longest LOS (mean 50 days). The 30 day mortality rate was 23.2%, with 61.5% of deaths attributable to groin and or stump infections. Reoperation (23%) correlated with a 30.7% mortality rate and significantly extended hospitalization.

**Conclusions:**

SSIs in vascular surgery, though relatively uncommon, are clinically significant, particularly following emergency groin or amputation procedures. The predominance of Gram-negative and poly microbial infections, including emerging enterococcus trends, highlights challenges in antimicrobial stewardship. Targeted prophylaxis, early detection and multidisciplinary management protocols are warranted for high-risk patients.

